# A Study of Disability Severity, Barriers, and Facilitating Factors in Accessing Healthcare Among Differently Abled Adults

**DOI:** 10.7759/cureus.75018

**Published:** 2024-12-03

**Authors:** Arunkumar M, Chidurala Rahul, Shriraam Karunakaran, Pankaj B Shah

**Affiliations:** 1 Community Medicine, Indira Medical College and Hospitals, Pandur, IND; 2 College of Medicine, Sri Ramachandra Institute of Higher Education and Research, Chennai, IND; 3 Biochemistry, MVJ Medical College and Research Hospital, Bangalore, IND; 4 Community Medicine, Sri Ramachandra Institute of Higher Education and Research, Chennai, IND

**Keywords:** among (disabled) differently-abled adults, assistive devices, disability care, government schemes, healthcare access, hearing impairment, musculoskeletal rehabilitation, public health, vision rehabilitation

## Abstract

Background: Disability impacts many individuals, thus restricting access to necessary healthcare. Barriers that affect health equity among people with disabilities include physical inaccessibility, financial constraints, and stigma in society. This study aims to report on the level of disability and factors determining healthcare access among adult differently abled persons in Chennai, Tamil Nadu, focusing on the WHO Disability Assessment Schedule (WHODAS 2.0).

Methods: In this cross-sectional study, 300 differently abled adults affiliated with the Tamil Nadu Udavikkaram Association participated. The severity of disability was assessed using WHODAS 2.0, while a structured questionnaire elicited details on socio-demographic characteristics, barriers to healthcare utilization, and facilitating factors. Data was analyzed using SPSS version 16 (IBM Corp., Armonk, New York, USA), with descriptive statistics and odds ratios calculated for key variables.

Results: Most participants (N=286, 93%) had mild levels of disability with problems mainly in self-care and mobility. The main barriers reported were healthcare expenses, distance to facilities, and lack of family support. Key facilitators were government schemes and assistive devices. Socio-economic factors like income and education showed marked correlations with the severity of disability.

Conclusion: Healthcare access varies with disability severity, revealing a need for targeted financial, familial, and accessibility interventions to reduce disparity. Future longitudinal studies may provide insights into the development of policies and programs to support individuals living with disabilities.

## Introduction

Differently abled populations face significant health disparities. Access to healthcare is restricted for people with disabilities (PWD) due to several physical, financial, communicational, and attitude barriers [1]. Identifying disability-specific barriers to healthcare service use is very important when intervention strategies and possible improvements in access to primary healthcare are used [2]. To bridge this gap, the present study has attempted to fill this gap by using the WHO Disability Assessment Schedule (WHODAS) questionnaire to estimate the severity levels of disability among those with different levels of disability (see Appendix, Sections A-D). This study also analyzes the factors influencing access to healthcare delivered by nongovernmental organizations (NGOs) in Chennai, Tamil Nadu. This study included disabilities like vision, hearing, and musculoskeletal or mobility disorders, as they were the most prevalent types according to previous studies [3,4]. The WHODAS scale was used to find the severity of disabilities. This will facilitate the association of disability severities with other factors. The study's primary goals were to determine the prevalence of various degrees of disability severity among adults with vision, hearing, and musculoskeletal impairments; to assess the barriers and facilitating factors that influence access to healthcare; to assess the health-seeking patterns of vision, hearing and musculoskeletal related differently abled adults; and to assess the associations between age, sex, educational status, marital status, total family income, barriers, adults who have varying degrees of disability severity and those who facilitate access to healthcare.

## Materials and methods

This study was a cross-sectional survey. Subsequently, those affiliated with an NGO, the Tamil Nadu Udavikkaram Association for differently abled, were approached; a total of 1500 differently abled individuals were registered, of which 300 were related to vision, hearing, and musculoskeletal-related differently abled, and all 300 differently abled adults were taken for study and gave consent to participate in this study. Research participants above 18 years of age with vision, hearing, and musculoskeletal disabilities were recruited. Permission from the NGO was sought. Before data collection, informed consent was obtained; the data were collected via a structured questionnaire that included detailed information about background characteristics, type of disability, questionnaires related to different levels of severity of disability among differently abled people, and questionnaires related to barriers and facilitators in accessing healthcare (see Appendix, Sections A-D). This sampling approach ensures diverse population representations and has been effective in similar studies on disability and healthcare access [5].

The study includes registered differently abled adults (over 18) who are NGO members for differently abled people, focusing on nine specific disability conditions outlined in the Rights of Persons with Disabilities Act of 2016. The remaining 12 disability conditions were excluded according to the Rights of Persons with Disabilities Act, 2016.

The study investigated the severity of disability among differently abled adults, focusing on outcomes such as disability severity classified as mild, moderate, or severe via the WHODAS 2.0 assessment and the barriers and facilitating factors influencing healthcare access. The permission to use this instrument was obtained from the World Health Organization. It investigated factors such as the type of disability (vision, hearing, or musculoskeletal) and access to healthcare services.

The predictors include age, sex, household income, educational status, relationship status, and health-seeking behavior. Potential confounders, such as economic status, academic level, and marital status, are considered because they can influence the severity of disability and access to healthcare. The study also identifies effect modifiers, such as the use of assistive devices and awareness of government programs, which may influence the relationship between disability severity and healthcare access.

**Figure 1:**
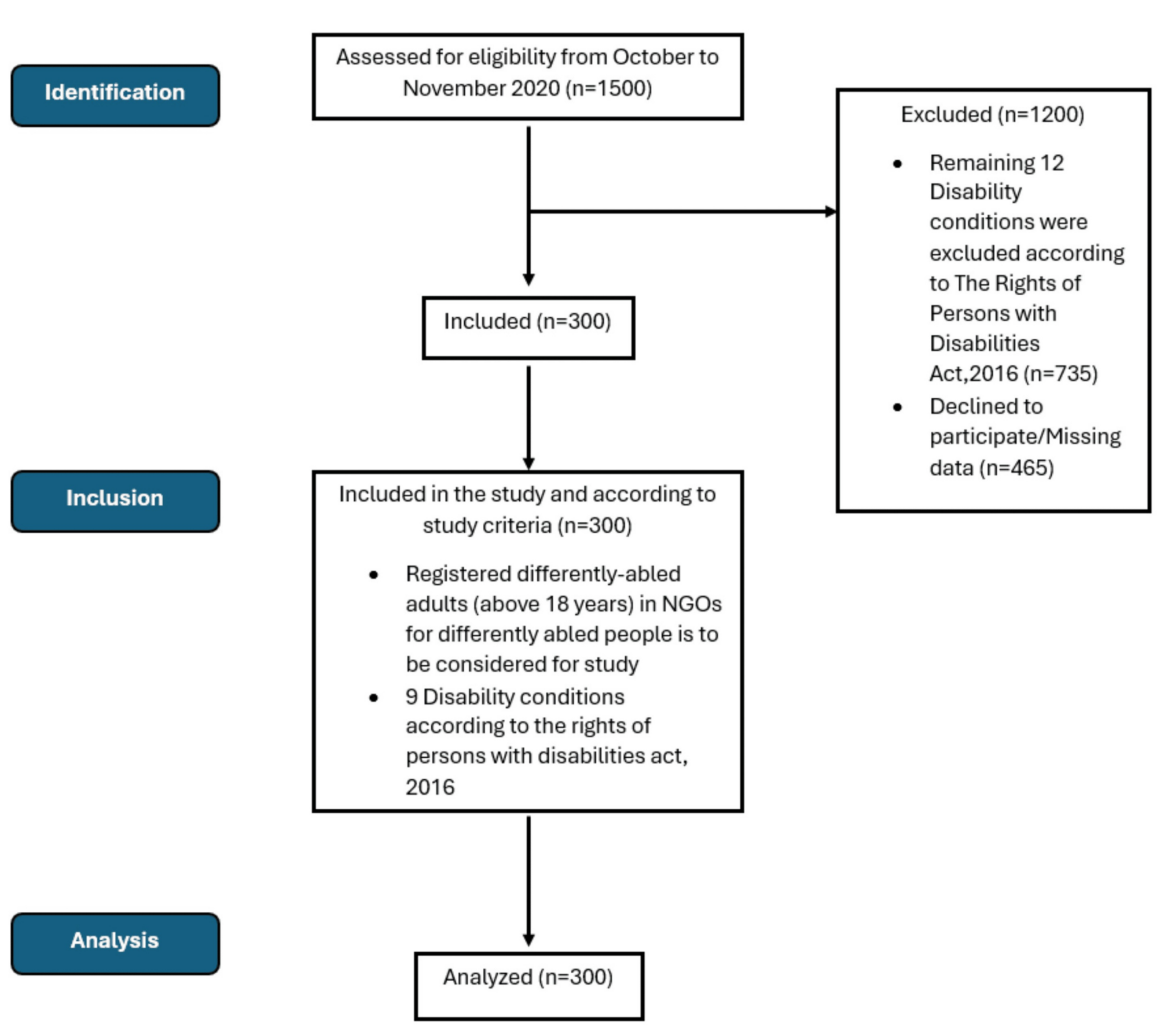
Overview of the study selection process.

Data input and analysis were done using the Statistical Package for Social Sciences (SPSS) version 16 software (IBM Corp., Armonk, New York, USA). Descriptive statistics were calculated for background characteristics, type of disability, prevalence of severity of disability, and health-seeking patterns among differently abled adults, which were calculated as percentages and 95% confidence intervals (95%CI). The odds ratio (OR) was calculated with a 95% confidence interval (CI) to assess the relationships between socio-demographic variables, type of disability, level of severity of disability, barriers, and facilitating factors in accessing healthcare. Mendeley reference management software (Elsevier, Netherlands) was used to cite the reference. The study selection process is shown in Figure 1.

## Results

**Figure 2:**
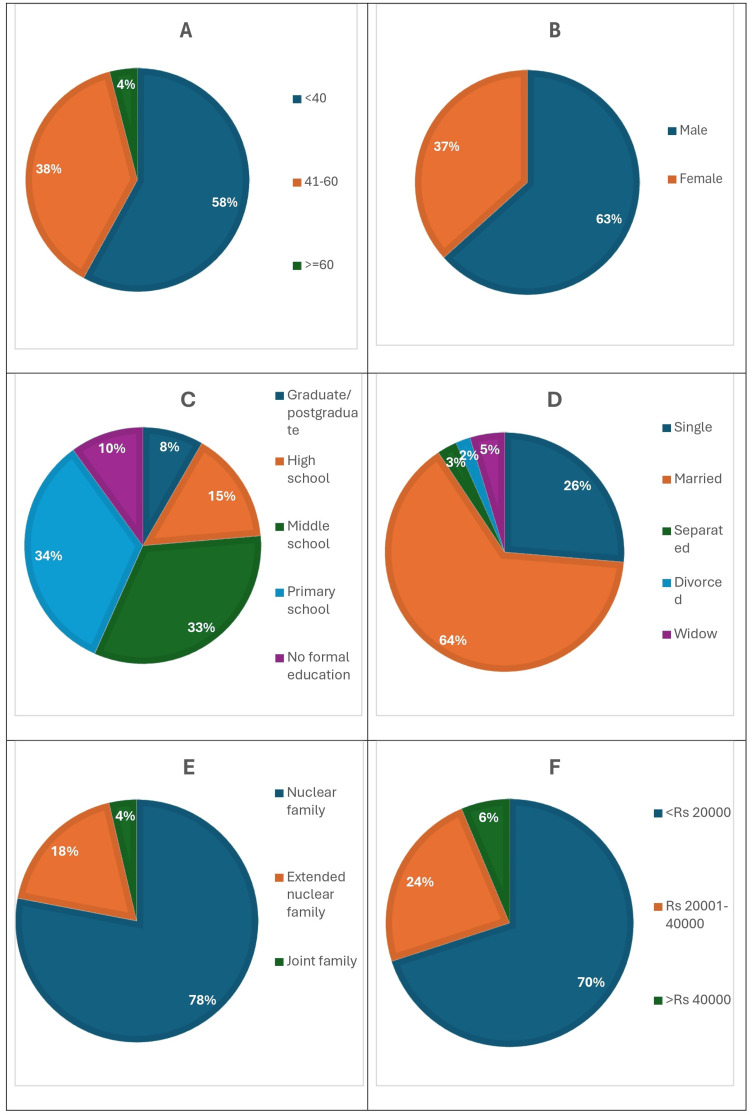
Patient characteristics. A: age; B: sex; C: education; D: marital status; E: type of family; F: income.

This study was conducted as a cross-sectional study in an NGO in Chennai, Tamil Nadu. In this study, 300 participants were from the 18-74 years age group. The mean age of the study participants was 40 years. Among the 300 participants, 174 (58%) were differently abled, belonging to the 18-40 years age group, 114 (38%) participants belonged to the 41-60 years age group, and only 12 (4%) participants belonged to the above 60 years age group. The majority of the participants were male, approximately 190 (63.3%), whereas 110 (36.7%) were female. In this study, 270 differently abled were literate, and only 30 participants, accounting for 10%, were illiterate. Among 300 participants, 100 (33.3%) completed at least primary school. Among the 300 participants, 234 (78%) were living in a nuclear family, and 210 (70%) were differently abled and had a monthly income of less than Rs. 20,000; of the majority of the differently abled participants, 221 (73.7%) were married, and 79 differently abled adults were unmarried. The details regarding the patient’s characteristics are shown in Figure 2.

The majority of participants were in the mild category in all domains, such as 76.7% (71.50-81.37) in getting around, 94% (90.68-96.41) in self-care, 88.3% (84.11-91.71) in getting along with people, 89.3% (85.24-92.56) in life activities at the household level and 90% (86.03-93.15) at the work or school level. In the domain of participation in society, 88.7% (84.56-92.05) were in the mild category. Among the 300 differently abled participants, 20 (6.7%) had a severe level of disability to get around. In the overall score, only 1.3% (0.35-3.33) had a severe level of disability, and 6% (3.59-9.32) had a moderate severity level.

Proportion of the level of severity in disability

Among those with vision-related disabilities, 70 had a mild severity level, seven had moderate, and only one had a severe level of disability. Among the 78 participants, 93.6% (90.21-96.09) had a mild level of disability in the domains of self-care and life activities at the household level. Approximately six participants, 7.7% (4.95-11.32), had severe levels of disability in the category of getting along with people. A total of 15.4% (11.51-19.99) of the participants had a moderate level of disability in the domain of getting around.

According to WHODAS, this study had about 71 (23.7%) hearing-related disabilities, with corresponding figures for mild, moderate, and severe levels of disability of 63 (88.7%), 8 (11.3%), and 0%, respectively.

In each domain, the majority of the participants were in the mild category: 66 (93%, 89.50-95.61) in self-care, 62 (87.3%) participants reported a mild level of participation in society and life activities at school/work, and 30 (10%) participants reported a severe level of disability in getting around and in household activities.

The prevalence of musculoskeletal-related disability was 151 participants (50.3%), and the corresponding figures of mild, moderate, and severe disability were 145 (96%), 3 (2%), and 3 (2%), respectively, according to the WHODAS. Among them, 144 (95.4%) had a mild category in the communication and understanding domain. Among them, 17 (11.3%) had severe levels of disability in getting around.

**Figure 3:**
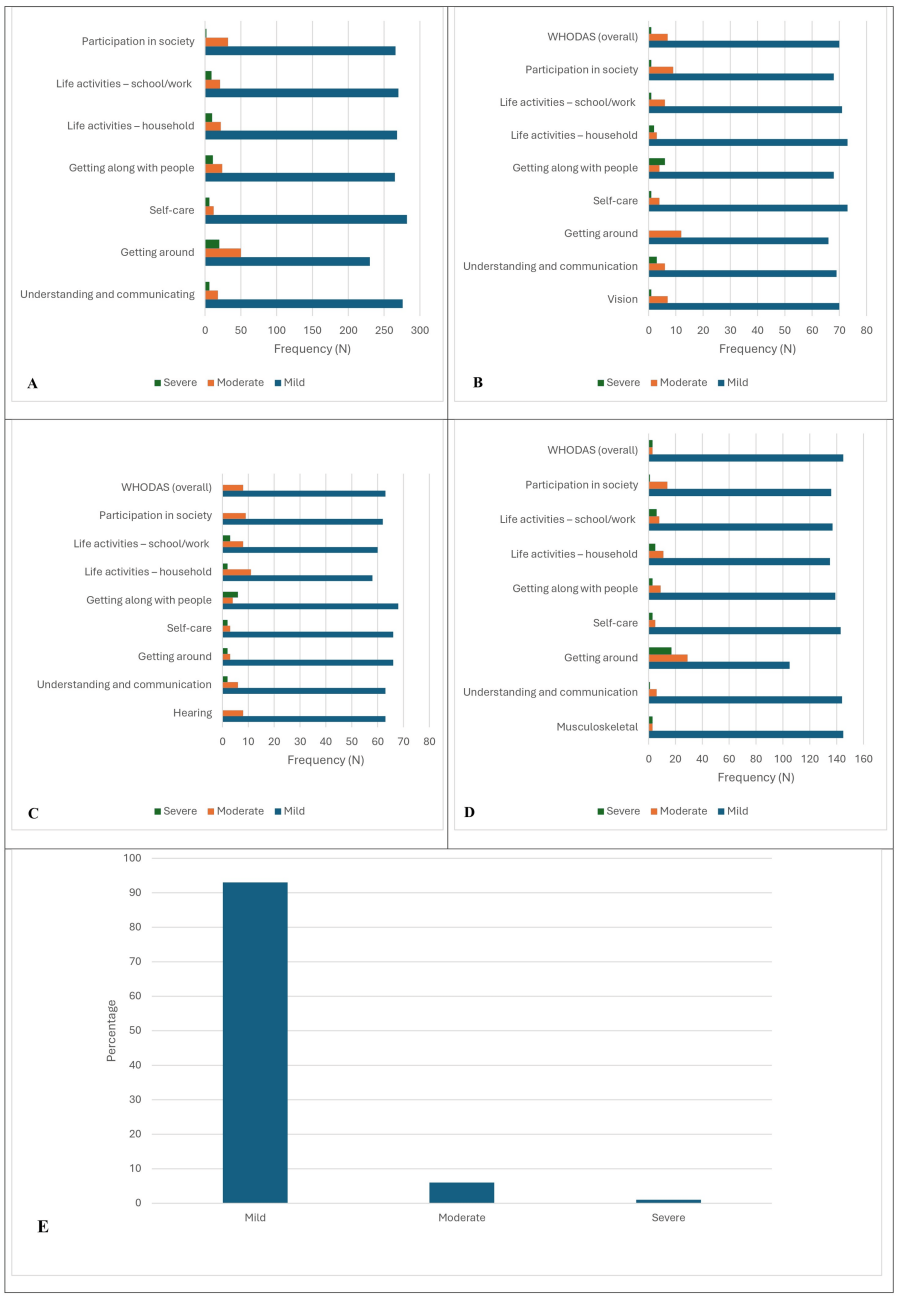
Levels of severity of disability. A: overall; B: vision; C: hearing; D: musculoskeletal; E: WHODAS (overall).

Twenty-nine participants (19.2%) had a moderate level of disability, whereas approximately 6 (4%) participants had a severe level of disability in life activities at school or work. The findings are summarized in Figure 3.

Barriers and facilitating factors in accessing healthcare

**Figure 4:**
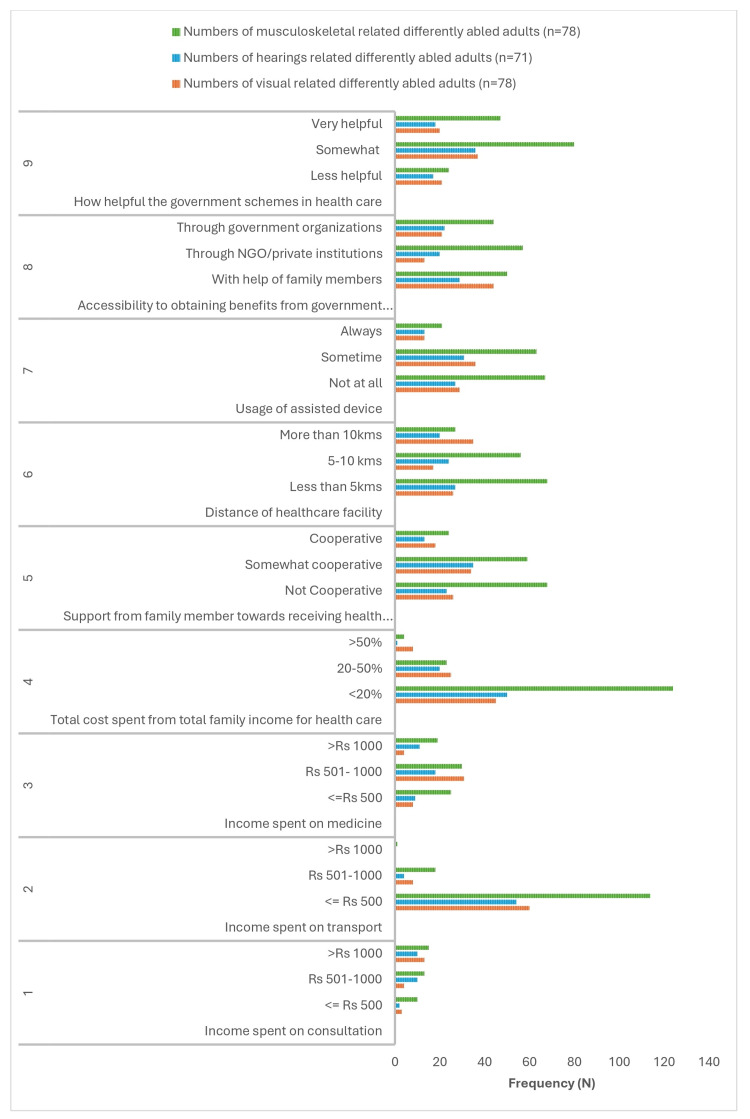
Barriers and facilitating factors.

Barriers and facilitating factors, such as the cost of consultation, transport, medication, and support from family members, government schemes, and assisted devices, influence access to healthcare. The results are shown in Figure 4.

Among the 78 vision-related differently abled adults, 13 (16.7%) participants spent more than Rs. 1000 for consultation in healthcare monthly, and approximately 66 (88.2%) participants spent less than Rs. 500 for transportation to healthcare services.

Forty-five participants (57.7%) had spent <20% of their total family income accessing healthcare, whereas 8 (10.2%) had spent more than 50% of their total family income. Forty-four participants (56.4%) accessed government schemes through family members' support, and approximately 36 (46.2%) participants used assisted devices sometimes. Thirty-five (44.9%) participants lived more than 10 km from the healthcare facility.

Among the 71 hearing-related differently abled adults, 50 (70.4%) participants spent less than 20% of their total family income on the cost of consultation, transport, and medicines in healthcare services. Approximately 27 (38%) participants had healthcare facilities within a distance of 5 km. Only 13 (18.3%) participants’ family members cooperated in supporting them in accessing healthcare. 

Thirty-one participants (43.7%) had used assisted devices for some time, and 13 (18.3%) had always used assisted devices. For 36 participants (50.7%), government schemes were somewhat useful in accessing healthcare.

The prevalence of musculoskeletal-related disorders was 151 (50.3%). Among those 15 (10.1%) participants who spent more than Rs.1000 on consultation, 114 (85.7%) had spent less than Rs. 500 on transportation in accessing healthcare, and 124 (82.1%) had spent <20% of the total family income on healthcare. The majority of the participants, 68 (45%), had support from their family members in accessing healthcare. Sixty-eight (45%) participants had healthcare facilities within a 5 km distance, and 67 (44.4%) had not used any of the assisted devices, only 21 (13.9%) had used assisted devices.

There were 57 (37.7%) participants who obtained the benefits of government schemes through NGOs or private institutions, and 80 (53%) utilized government healthcare schemes, which were helpful.

Association of certain risk factors with level of severity in disability

Table 1 shows the associations among age, sex, education status, marital status, total family income, barriers and facilitating factors in accessing healthcare, and level of severity of disability among vision-related differently abled adults.

**Table 1:** Associations of specific risk factors with level of severity of disability.

Risk factors		Levels of severity in disability in visually related differently abled adults			Levels of severity in disability among hearing-related differently abled adults			Levels of severity in disability among musculoskeletal-related differently abled adults		
		Moderate and severe	Mild	Odds ratio	Moderate and severe	Mild	Odds ratio	Moderate and severe	Mild	Odds ratio
Age in years	≥40	28	33	2.5	4	41	1.86	4	63	2.6
	<40	42	45		4	22		2	82	
	Total	70	78		8	63		6	145	
Sex	Female	33	37	1.12	6	17	8.12	4	97	1.01
	Male	37	41		2	46		2	48	
	Total	70	78		8	63		6	145	
Educational qualification	Literate	58	66	0.879	8	54	0.87	5	137	3.4
	Illiterate	12	12		0	9		1	8	
	Total	70	78		8	63		6	145	
Marital status	Married	50	55	0.66	8	42	1.19	5	30	0.05
	Unmarried	20	23		0	21		1	115	
	Total	70	78		8	63		6	145	
Total family income		53	59	1.03	7	46	0.38	4	94	0.92
	≥Rs 20,000	17	19		1	17		2	51	
	Total	70	78		8	63		6	145	
Income spent on consultation	No	53	58	0.53	7	42	3.5	3	35	0.31
	Yes	17	20		1	21		3	110	
	Total	70	78		8	63		6	145	
Income spent on transport	Yes	6	10	10.66	5	53	3.18	5	128	1.5
	No	64	68		3	10		1	17	
	Total	70	78		8	63		6	145	
Income spent on medicine	No	30	35	2.22	7	26	9.96	5	70	0.18
	Yes	40	43		1	37		1	75	
	Total	70	78		8	63		6	145	
Support from family members	Cooperative	45	52	0.25	5	43	1.29	5	78	0.23
	Not cooperative	25	26		3	20		1	67	
	Total	70	78		8	63		6	145	
Distance of healthcare facility	More than 5 km	46	52	1.56	7	37	4.91	3	65	0.81
	Less than 5 km	24	26		1	26		3	80	
	Total	70	78		8	63		6	145	
Government schemes are helpful	No	17	20	1.87	6	12	12.75	5	42	12.26
	Yes	53	58		2	51		1	103	
	Total	70	78		8	63		6	145	
Assisted device	Not at all	43	49	1.88	6	21	0.16	5	62	0.14
	Always	27	29		2	42		1	83	
	Total	70	78		8	63		6	145	

Compared with those in the illiterate group, those in the literate group were less at risk of having a severe level of disability with an odds ratio of 0.87 (0.80-0.97), and the difference was statistically significant.

Among the vision-related differently abled, the individuals who have not spent income on transport to a healthcare facility were found to have 10.6 (2.11-53.84) times greater odds of having a severe level of disability than those who have spent income on transport to a healthcare facility, and the difference is statistically significant.

Table 1 shows the associations among age, sex, education status, marital status, total family income, barriers and facilitating factors in accessing healthcare, and level of severity of disability among hearing-related differently abled adults. In the present study, there were 8.11 (1.49-44.18) times more female hearing-related disabled adults at risk of having a severe level of disability when than male hearing-related disabled adults, and the difference was found statistically significant.

Among the study participants, those who were married had a greater risk of having a severe level of disability than unmarried, differently abled ones, with an odds ratio of 1.19 (1.05 -1.34), and the difference was statistically significant.

Among hearing-related differently abled, the individuals who have not spent income on medication are found to have 9.96 (1.15-85.90) times greater odds of having a severe level of disability than those who have paid income on medicines, and the difference is statistically significant. In the present study, among the hearing-related differently abled, those who had not utilized government schemes were 12.7 (2.28-71.16) times more likely to have a severe level of disability than were the participants who utilized government schemes, and this difference was statistically significant.

Among hearing-related differently abled, the individuals who had always used assisted devices were 0.16 (0.03-0.90) times less likely to have a severe level of disability than those who had not used assisted devices, and the difference was statistically significant.

Table 1 shows the associations of age, sex, education status, marital status, total family income, barriers and facilitating factors in accessing healthcare, and level of severity of disability among musculoskeletal-related differently abled adults. Among the study participants, those who were unmarried had a lower risk of having a severe level of disability than married, differently abled adults with an odds ratio of 0.05 (0.01-0.47), and the difference was statistically significant. In the present study, among the musculoskeletal-related differently abled, those who had not utilized government schemes were 12 (1.40-108.12) times more likely to have severe levels of disability than were the participants who utilized government schemes, and this difference was statistically significant.

Health-seeking pattern among the differently abled adults

**Figure 5:**
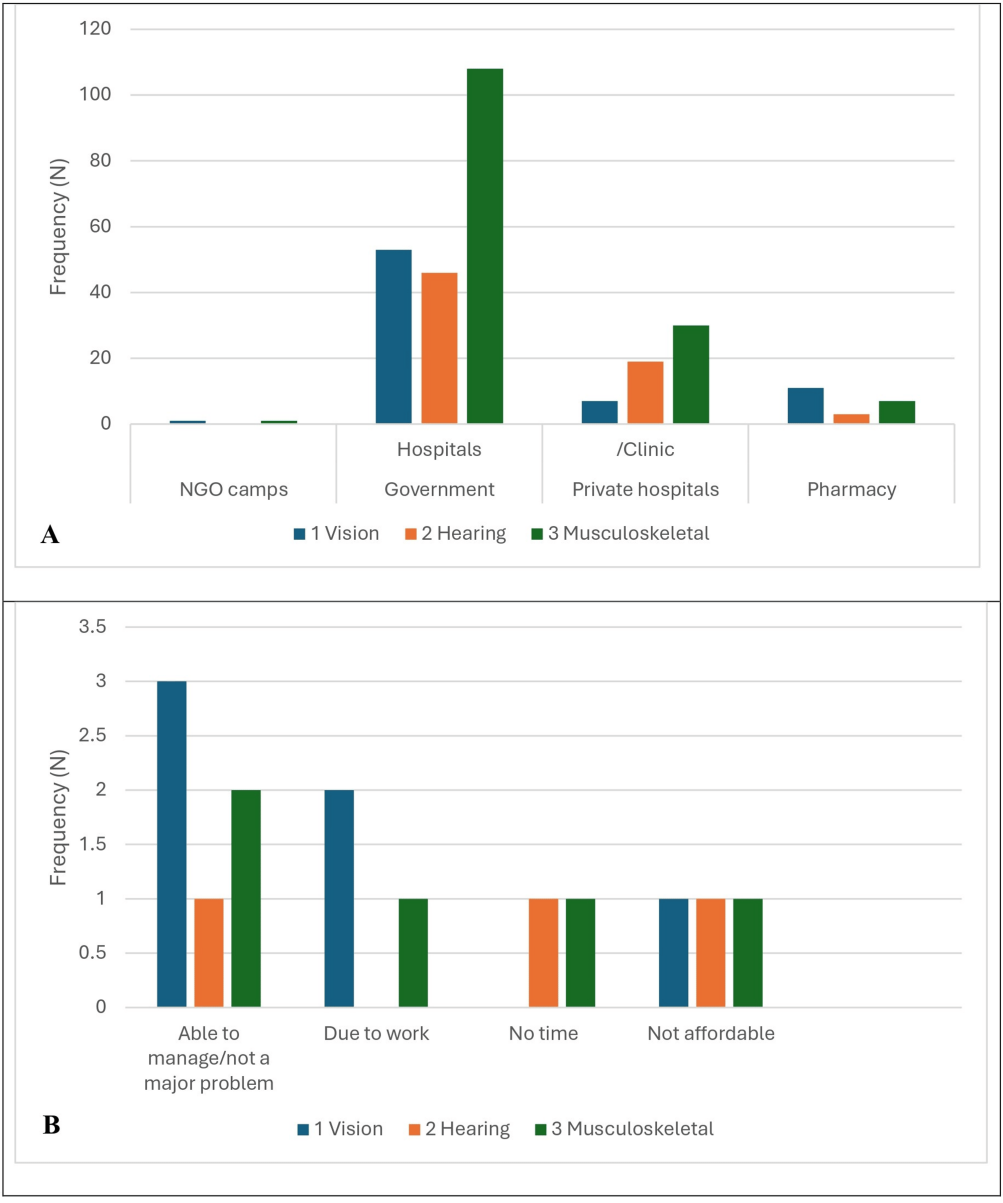
Health-seeking patterns among the differently abled adults. A: Type of health facility approached for the health problems; B: reason for not taking treatment for health problems.

The health-seeking behavior of the differently abled in the NGOs during the last 12 months is shown in Figure 5.

In this study, 286 out of 300 participants sought medical attention for their health condition: 72 (92.3%) for vision, 68 (95.7%) for hearing, and 146 (96.6%) for musculoskeletal-related differently abled.

Most participants, 53 (67.9%), 46 (64.8%), and 107 (71.5%), sought healthcare from government hospitals in vision, hearing, and musculoskeletal categories, respectively. Thirty (19.9%) musculoskeletal-related differently abled adults sought healthcare from private hospitals or clinics.

Among the 300 participants, only 14 (4.7%) had not sought medical attention, and the majority had vision-related disabilities. The most common reason was "Able to manage or not a major problem," which was said to be visually disabled 3 (3.8%). A total of 22 (1.3%) participants said that the reason was "Not affordable" and "No time" by hearing and musculoskeletal-related differently abled adults. Approximately 286 (94%) participants sought medical attention, while 206 (68.7%) were taken to hospitals from government hospitals.

## Discussion

The present study attempted to evaluate the severity of disability and barriers to and facilitate factors for healthcare accessibility among differently abled adults in Chennai, Tamil Nadu. The WHODAS 2.0 assessment provides essential insights into the prevalence of different severities of disability and the factors affecting access to healthcare in this population. When used alone, there are components of scales that can diminish the prevalence, and when used together, as in WHODAS 2.0, it can exaggerate the prevalence. Hence, a precise estimation of the burden of disabilities within a defined geographical area is vital. In the present study, 300 participants aged 18-74 years were included.

The results indicated that, in general, most of the differently abled participants were mildly disabled in all the WHODAS 2.0 domains. However, substantially high proportions had problems with self-care and getting around. The results agree with the general trend globally that the burden of disability is predominantly mild, although variations depend on the specific domain under study [6-9]. This is in domains such as life activities and social participation, where mild severity is observed. Target-oriented interventions are needed to help this group maintain functional independence. After adjusting for potential confounders, the probability of not seeking care was more significant in people who reported perceived discrimination [10]. These comparisons revealed that differently abled individuals engaged in more health-seeking behaviors in both studies. In another study conducted in Bangladesh, the researchers revealed the reasons the participants revealed for consulting a formal healthcare provider over an informal healthcare provider included specialized (96%) and good quality (74%) care, which they felt they could only get from a formal healthcare provider [11]. Easy access (63%) was another critical factor that led them to seek treatment from a formal healthcare provider [12].

It also identified key barriers to healthcare access: financial constraints, long physical distance to healthcare facilities, and poor support from families. In keeping with this, less than half of the participants spent less than 20% of their family income on healthcare. This would suggest that although there are financial barriers, they may not be as significant as other barriers for this population. This contrasts with the findings of studies in different regions that suggest that financial barriers are an essential factor [13].

A systematic review revealed that the different criteria, limitation parameters, and cut-off values used in other studies, which are equally valid, are highly varied, rendering the results incomparable [14]. Previous studies have shown that, very often, there is an inverse relationship between the severity of a disability and educational attainment and income level [15]. In this study, the results show that, among the differently abled adults, higher schooling and income levels are inversely related to severe disability, therefore highlighting the protective role of socio-economic factors. At higher levels of education, lower levels of frailty and disability were observed. Education is a protective factor against frailty and disability in China, India, Russia, and South Africa [16]. Compared with the current study, the results of education are comparable in terms of protective factors for disability. After adjusting for potential confounders, the probability of not seeking care was more significant in people who reported perceived discrimination. These comparisons revealed that differently abled individuals engaged in more health-seeking behaviors in both studies. A study in Nigeria reported contradictory results concerning healthcare-seeking outcomes, with 28.5% of respondents not seeking healthcare, while 41.5% sought care only once during their last three illness episodes [17]. Additionally, more than half (56.3%) reported experiencing unmet healthcare needs [18]. A study reported that only 50% of participants with disabilities sought some form of medical treatment. Furthermore, the study highlights that treatment-seeking was neglected and ignored, leading to a severe degree of disabilities [19]. Musculoskeletal or mobility disorders have a larger impact on the prevalence of diabetes, hypertension, and other central nervous system-related disorders [20-22].

The findings on the relationship between marital status and disability severity complicate this picture [14]. Although generally, married participants tend to have lower levels of disability severity, the data suggest that such a protective effect may vary depending on the type of disability, for example, musculoskeletal disabilities show less of a marital status gradient. This complexity is mirrored in studies from other regions where the impact of marital status on disability outcomes varies [23-25].

It also identified the use of assistive devices and access to government schemes as strong facilitators of reducing disability severity [12,22]. Indeed, participants who consistently used assistive devices or received support from the government had lower odds ratios for experiencing severe disability. This, in turn, supports broader literature, which has acknowledged the role of assistive technologies and social support systems in mitigating the effects of disability.

One of this study's key strengths is that it operationalizes the severity of a disability through multiple domains via the WHODAS 2.0 tool, which is more standardized in terms of cultural and environmental contexts. In addition, a varied sample from different NGOs increases generalizability to the population of differently abled adults in Chennai. These research results are informative and helpful for health professionals and policymakers. The degree of mild disability is sufficiently high that early intervention and prevention are expected to become more critical elements in preserving functional independence among the differently abled adult population. Such a system needs to orient itself toward being accessible and affordable, especially when discussing assistive devices and special services, as these have been salient barriers identified. Moreover, policymakers should extend and improve government schemes for differently abled persons. Community-based programs that include education and engaging family members may effectively improve health for this population since family support plays a vital role in gaining easy access to healthcare. Further research is needed to determine how such barriers and facilitators of evidence-based care may affect progressive disability or health outcomes over time. The study highlights the complex interplay between the level of disability, social class, and healthcare available, which differently abled adults in the city of Chennai face. It identifies barriers and facilitators to accessing healthcare. There will be worthwhile lessons in doing this for targeted interventions to reduce inequity for this vulnerable population. Long-term impact studies on these variables, and targeted strategies for accessible, inclusive healthcare could be planned for all.

However, this study has several limitations. Since the participants were only a sample drawn from the Tamil Nadu Udavikkaram Association, the sample may not represent the larger population, especially for those not belonging to the NGO. The study was conducted within the limits of Chennai in Tamil Nadu, and the findings may not be generalizable to the rural or other urban areas having different healthcare infrastructure and socio-economic factors. The study might not consider every cultural factor that affects attitudes relating to disability and healthcare access, which may be highly variable across different parts of India. Because of the cross-sectional design, the severity of disability and identified barriers and facilitators cannot be inferred to have a causal relationship. Moreover, with over-reliance on self-reported data in virtually all measures of healthcare access and health-seeking behavior assessment, the potential for response bias is high. Future studies should incorporate longitudinal designs and objective measures of health outcomes.

## Conclusions

In conclusion, this study reveals many barriers and facilitators regarding access to healthcare for differently abled adults in Chennai. Most of the respondents had mild levels of disability, but financial restrictions, distance of facilities, and poor family support were the major barriers. However, government schemes and assistive devices are valuable resources that, to a very large extent, enhance people's access and quality of life. More focused interventions, policy reforms, and community-based support nowadays emphasize the importance of bridging gaps for healthcare access in health equity for the abled and disabled alike.

## Figures and Tables

**Figure 6:**
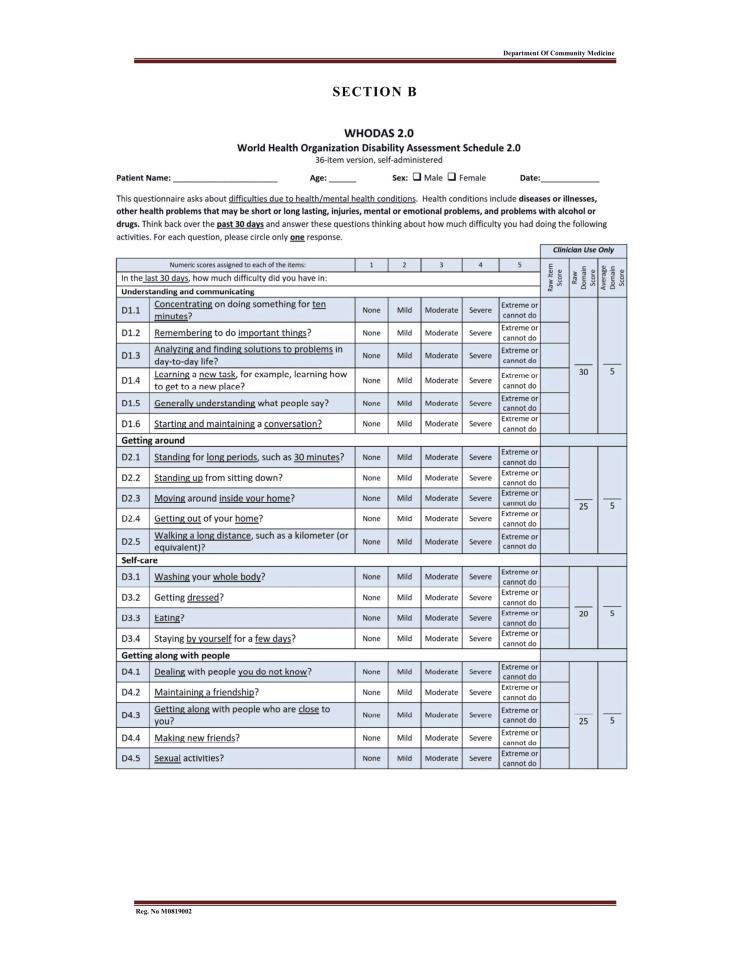
WHODAS 2.0 Questionnaire page 1.

**Figure 7:**
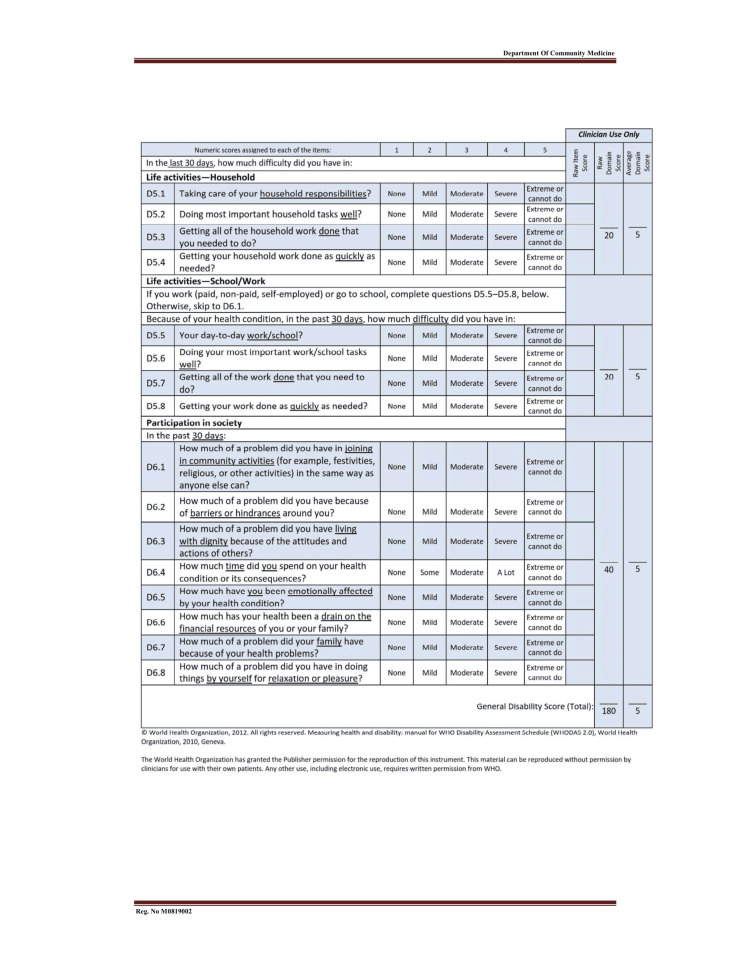
WHODAS 2.0 Questionnaire page 2.

## Appendices

Section A: Socio-demographic profile

**Table 2:** Background characteristics.

S. No	Question	Response
1	Name	
2	Age (completed years)	
3	Sex	Male/female
4	Residential address:	
5	Educational qualification	Graduate or postgraduate/intermediate or post-high school diploma/high school/middle school/primary school/illiterate
6	Marital status	Single/married/separated/divorced/widow/widower
7	Type of family	Nuclear/extended nuclear/joint/others
8	Family details	Name/age/sex/education/occupation/income/relation to worker
9	Total family income per month	
10	Socio-economic class (modified BG Prasad classification)	Class I/Class II/Class III/Class IV/Class V
11	Type of disability	Vision/hearing/musculoskeletal

Section B: WHODAS 2.0 Questionnaire

Section C: Question to assess barrier and facilitating factors

**Table 3:** Question to assess barrier and facilitating factors in accessing healthcare among differently abled.

Q. No.	Question/statement	Options
1	Income spent on transport-related to healthcare	Open-ended
2	Income spent on medicine	Open-ended
3	Income spent on transport-related to healthcare	Open-ended
4	How much is the cost spent from total family income for transport, drugs, and services for healthcare?	a) <20%, b) 20%-50%, c) >50%
5	How do the other family members cooperate in providing healthcare?	a) Not cooperative, b) Somewhat cooperative, c) Cooperative
6	How far is your nearest healthcare facility?	a) Less than 5 km, b) 5-10 km, c) more than 10 km
7	Are you using any assisted device?	a) Not at all, b) Sometimes, c) Always
8	How did you obtain your benefits from Government schemes like pensions?	a) With help of family members, b) Through NGO/private institutions, c) Through government organizations
9	How helpful are government schemes and programs in healthcare for the differently abled?	a) Less helpful, b) Somewhat helpful, c) Very helpful

Section D: Health-seeking behavior among differently abled

**Table 4:** Health-seeking behavior.

S.no	Problems	Whether sought healthcare services	
		Yes	No
1	Vision		
2	Hearing		
3	Musculoskeletal		

**Table 5:** Health-seeking behavior part 2. NGO: nongovernmental organization, GH: government hospital, PHC: primary health center.

S.no	Problems	Where do you seek healthcare for your problem?				
		NGO	GH/PHC	Private clinic	Pharmacy	Others
1	Vision					
2	Hearing					
3	Musculoskeletal					
